# Prevalence of susceptibility to *Cryptosporidium* spp. among dairy calves with different feeding regimens with an emphasis on the feeding of transition milk

**DOI:** 10.14202/vetworld.2022.1256-1260

**Published:** 2022-05-22

**Authors:** Alīna Zolova, Dace Keidāne, Maksims Zolovs

**Affiliations:** 1 Faculty of Veterinary Medicine, Institute of Food and Environmental Hygiene, Latvia University of Life Sciences and Technologies, Jelgava, Latvia; 2 Department of Biosystematics, Institute of Life Sciences and Technology, Daugavpils University, Daugavpils, Latvia; 3 Statistics Unit, Riga Stradins University, Riga, Latvia

**Keywords:** calves, colostrum, *Cryptosporidium*, neonatal diarrhea, transition milk

## Abstract

**Background and Aim:**

Colostrum composition and importance for newborn organisms were repeatedly studied. However, the interest in transitional milk usefulness is weak and recommendations concerning transition milk intake are not developed. The aim of this study was to evaluate whether transition milk intake after colostrum consumption affects the chances of calf infection with *Cryptosporidium* spp.

**Materials and Methods:**

We collected data for *Cryptosporidium* spp. infection from calves (n=425) divided into three groups: The first group – supervised colostrum and transition milk intake; the second group – supervised colostrum and whole milk intake; and the third group – not supervised colostrum and whole milk intake. To detect oocysts of *Cryptosporidium* spp. in feces, the flotation method was used, and slides were stained using the modified Ziehl-Neelsen method. Generalized linear mixed modeling was conducted to determine whether the explanatory variable – the management of colostrum and transition milk feeding with three categories (three research groups) – was related to the probability of calves incurring infection with *Cryptosporidium* spp.

**Results:**

In the first group, 26.1% of calves were positive for the presence of *Cryptosporidium* spp. oocysts, in the second – 37.2%, and in the third – 44.1%. Statistical data analysis showed that calves who did not receive transition milk after colostrum consumption had increased chances of having *Cryptosporidium* spp. (by 1.90-2.47 times on average). The main results showed that the management of colostrum and transition milk feeding is related to *Cryptosporidium* spp. infection, indicating that both colostrum and transitional milk play a significant role in controlling pathogenic infections.

**Conclusion:**

The most effective management of colostrum and transition milk feeding against *Cryptosporidium* spp. infection is the timely intake of an adequate amount of colostrum followed by transitional milk consumption for at least 2 weeks before weaning from the dam.

## Introduction

Neonatal diarrhea is a hazardous condition responsible for calf mortality worldwide. Several pathogens (Cryptosporidium, Escherichia coli, Rotavirus, Coronavirus, and Coccidia) cause diarrhea in young calves, with *Cryptosporidium* being the most common pathogen found in calves. It is a microscopic parasite that can cause a disease called cryptosporidiosis. The disease decreases the absorption of essential nutrients from milk and results in weight loss, dehydration, or even calf death [1-3]. The treatment of cryptosporidiosis is difficult as there are no vaccines available to prevent the disease, while available drugs often focus only on symptomatic (such as dehydration) treatment [4,5]. Therefore, effective farm management has become an alternative instrument for controlling or preventing cryptosporidiosis in livestock. For example, frequent removal of feces from cowsheds and calves’ pens, as well as applying disinfectants, hot water, and desiccation may help to markedly reduce the number of oocysts in the environment [6,7].

Another potential method of cryptosporidiosis control is the management of colostrum and transition milk feeding. Since calves are born naively immune, they need the support that cows provide by producing colostrum and transitional milk. Colostrum is the first milk that cows produce after calving. It is a complex secretion that contains markedly elevated amounts of essential chemical compounds (nutrients, growth factors, and immune factors), which aim to contribute to the immune system and feed the newborn calf [8-10]. Following the first milking, cows produce transition milk that may stimulate development of the gastrointestinal tract [11]. In practice, no clear line can be drawn regarding when colostrum is transformed into transitional milk and then into whole milk.

Based on recent research regarding evidence of colostrum composition and importance for newborn organisms, some recommendations for colostrum allowances and timely feeding of newborn calves were developed [12,13]. However, the interest in transitional milk usefulness is weak and recommendations concerning transition milk intake are not developed. For example, on large dairy farms, the calves are weaned from the cows and kept separately in the calves’ pen soon after the first feeding, while the transitional milk is placed in a common storage tank where it is diluted with other milk.

This study aimed to test the association between *Cryptosporidium* spp. infection and the management type of colostrum and transition milk feeding in calves.

## Materials and Methods

### Ethical approval

All procedures performed in the study involving animals were in accordance with ethical standards. The study was approved (No. DzAĒP/2017/2) by the Animal Welfare and Ethical Council of the Faculty of Veterinary Medicine, Latvia University of Life Sciences and Technologies, and complied with current laws in Latvia.

### Study period and location

The study was conducted from December 2018 to December 2020. All coprological samples were examined on the collection day. Laboratory examinations were made in the Laboratory of Parasitology, Institute of Food and Environmental Hygiene, Faculty of Veterinary Medicine, Latvia University of Life Sciences and Technologies.

### Sample collection and examination

**Figure-1:**
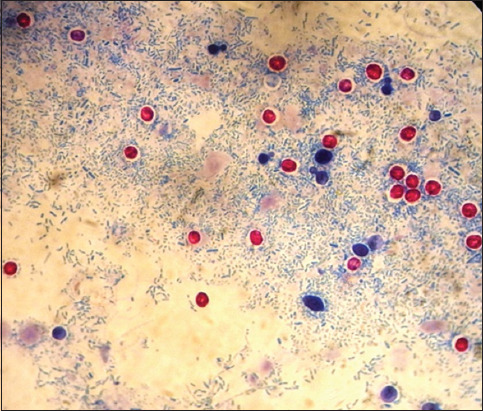
Oocysts of Cryptosporidium spp. stained with the modified Ziehl-Neelsen method.

Fecal samples of calves were collected in disposable polyethylene packages and stored in a transportable cooler during transport to the laboratory until examined. To detect oocysts of *Cryptosporidium* spp. in feces (Figure-1), the flotation method was used according to Fujino *et al*. [14]. Slides were stained using the modified Ziehl-Neelsen method [15]. Samples for microscopy were previously prepared using the saturated NaCl flotation method. For the flotation, 1 g of fecal sample was used and after continuous flotation and centrifugation steps, it resulted in 2 mL of concentrated material, which was used for further analyses.

### Calves feeding regimens

Overall, 425 calves (15±2 days old) from 39 farms were examined in this study: The first group – calves (n=153) received colostrum of ~2.5 L within the first 0-4 h of life (supervised or assisted where necessary – calves were fed immediately after they were born), ~4 L within the first 12 h of life and then continued receiving transition milk within 2 weeks from their dams; the second group – calves (n=145) received colostrum of ~2.5 L within the first 0-4 h of life (supervised or assisted where necessary – calves were fed immediately after they were born), ~4 L within the first 12 h of life and then weaned from the dams and kept separately by feeding milk from a common storage tank where the dams’ transition milk was diluted with other milk; and the third group – calves (n=127) were born at night and there was no certainty that a sufficient amount of colostrum was ingested on time; these calves were weaned from the dams and kept separately by feeding milk from a common storage tank where the dams’ transition milk was diluted with other milk.

The number of cows on one farm ranged from 3 to 300 head. The sample was not limited by calving season, the length of the dry period, cow breed, or age. No dry cow vaccination against rotavirus-, coronavirus-, and *E. coli* bacteria was done. Calves were housed in individual pens. The first research group received milk from cows on demand, whereas the second and third groups received milk from a bucket with 4 feeding times during the 1^st^ week and 3 feeding times during the 2^nd^ week.

### Statistical analysis

Generalized linear mixed modeling was conducted to determine whether the explanatory variable – the management of colostrum and transition milk feeding with three categories (three research groups) – was related to the probability of calves’ incurring infection with *Cryptosporidium* spp. where the farm identification number (“FarmID”) was set as a random effect variable. The prevalence of parasites was calculated as the percentage of hosts infected by *Cryptosporidium* spp. Statistical data analysis was conducted using Jamovi, version 2.0.0 [16].

## Results

Out of all the fecal samples analyzed (n=425), 35.3% of calves were positive for the presence of *Cryptosporidium* spp. oocysts. The presence of clinical signs of diarrhea was recorded in 20.6% of the positive animals**.** The percentage of calves positive for the presence of *Cryptosporidium* spp. oocysts and the percentage of calves with the presence of clinical signs of diarrhea in each research group are summarized in Table-1. The regression model was significant (χ^2^ (2)=8.62, p=0.0013), indicating the variance in the chance of incurring one of the two outcomes of *Cryptosporidium* spp. infection is explainable by the management of colostrum and transition milk feeding. The second research group significantly predicted the chance of incurring infection by *Cryptosporidium* spp. (B=0.643, z=2.00, p=0.046, OR=1.901). This indicates that calves belonging to the second research group had an increased chance of having *Cryptosporidium* spp. by 1.90 times on average (95% CI [1.012-3.574]) compared to those of the first research group. The third research group significantly predicted the chance of infection with *Cryptosporidium* spp. (B=0.901, z=2.93, p=0.003, OR=2.469). This indicates that calves belonging to the third research group had increased chances of having *Cryptosporidium* spp. by 2.47 times on average (95% CI [1.35-4.52]) compared to those of the first research group.

**Table 1:** The percent of positive calves for the presence of *Cryptosporidium* spp. oocysts and the percent of calves with the presence of clinical signs of diarrhea.

Research groups	Diarrhea (%)	Positive (%)	Negative (%)
The first research group (timely colostrum+transition milk)	15	26.1	73.9
The second research group (timely colostrum+whole milk)	46.3	37.2	62.8
The third research group (not supervised intake of colostrum+whole milk)	48.2	44.1	55.9

## Discussion

The results showed that the management of colostrum and transition milk feeding is related to *Cryptosporidium* spp. infection, indicating that both colostrum and transitional milk play a significant role in controlling pathogenic infection. The most effective combination against *Cryptosporidium* spp. infection is the timely intake of an adequate amount of colostrum followed by transitional milk consumption for at least 2 weeks before weaning from the dam.

Our results for *Cryptosporidium* oocyst shedding (average prevalence=35.3%, from 26.1% to 44.1%) were similar to reported results from Estonia, prevalence=30% [17] and prevalence=23% [18]; however, markedly different compared to Lithuania (prevalence=67%) [19], which was explained mainly by the small sample size in Lithuania.

The protective properties of colostrum were repeatedly studied and are mainly explained by transfer of immunoglobulins (such as IgG) from the dam to the calf through the ingestion of colostrum [20]. However, Derbakova *et al*. [21] did not record a relationship between the level of IgG in bovine colostrum and the likelihood of *Cryptosporidium* spp. infection in calves and suggested that innate and adaptive immunity play more significant roles in immune responses to *Cryptosporidium* species than mother passive transfers of immunity to the offspring. Our research findings show that the timely ingestion of an adequate volume of colostrum plays a significant role in the likelihood of *Cryptosporidium* spp. infection in calves, suggesting that the role of other bioactive compounds of colostrum (growth factors, hormones, cytokines, enzymes, polyamines, nucleotides, antimicrobial components, white blood cells, etc.) should not be underestimated. Moreover, the IgG and other immune compounds should be considered as one complex system where several elements cooperate with each other to create one universal barrier against pathogens, as there is evidence of coinfection with other viral and bacterial pathogens [3]. In addition, the results of our study (the increased percentage of diarrhea) indirectly showed that colostrum intake should be monitored so that it is ingested immediately after birth, which significantly reduces the chances of pathogenic infection. An explanation for these findings may be found in Fischer *et al*. [22], who demonstrated that calves fed immediately after birth (0 h) had greater serum IgG concentrations compared with calves fed at 6 and 12 h after birth, as well as influencing the establishment of the calf gut microbiome [23].

The results of our research show that calves fed transition milk for at least 2 weeks following the colostrum meal have significantly lower chances of having *Cryptosporidium* and experiencing diarrhea compared to calves receiving colostrum and then whole milk. These results are supported by Conneeley *et al*. [24] and Kargar *et al*. [25], who investigated the health status of calves fed transitional milk. For example, Kargar *et al*. [25] showed that extending the duration of feeding transitional milk positively influenced calf weight gain and decreased the chance of having diarrhea. This is explained by the greater concentrations of some bioactive compounds in transitional milk compared with whole milk [9,22,26].

The skipping of transitional milk ingestion is always associated with the weaning process, where the calf undergoes multiple stressful situations. For example, a calf not weaned from the dam receives milk on demand in unlimited quantities. In contrast, a weaned calf undergoes a specific feeding program that may not match the individual physiological needs of the calf. This influences the development of the gastrointestinal system, which serves as the first barrier to infections [27]. The other stressful factor is moving the animal to a calf pen. It will live in a limited area exposed to other different aged calves (with different health statuses and infections), while its immune system remains naïve. Intensive farming management has some evolutionary implications for parasites and pathogens, as discussed by Mennerat *et al*. [28]. For example, the high density of hosts in a limited territory affects the probability of parasite transmission stages to contact new hosts and faster parasite development and increases the probability of coinfections [29,30]. In addition, the gut microbiota undergoes changes [31] during the weaning process, which may affect the predisposition to diarrhea. The weaning strategy can result in great differences in growth performance, gastrointestinal development, and health status; therefore, it should be chosen carefully to minimize economic losses.

## Conclusion

The most effective management of colostrum and transition milk feeding against *Cryptosporidium* spp. infection is the timely intake of an adequate amount of colostrum followed by transitional milk consumption for at least 2 weeks before weaning from the dam. Colostrum feeding can be cross-checked by estimating the serum refractometry to identify the phenomenon of failure of passive transfer.

## Data Availability

The supplementary data can be available from the corresponding author on a reasonable request.

## Authors’ Contributions

AZ: Study design and collected and examined the samples. DK: Supervised the research process. MZ: Collected samples and performed statistical analysis. AZ and MZ: Wrote the manuscript with input from all authors. All authors read and approved the final manuscript.

## References

[1] Brainard J, Hooper L, McFarlane S, Hammer C.C, Hunter P.R, Tyler K (2020). Systematic review of modifiable risk factors shows little evidential support for most current practices in *Cryptosporidium* management in bovine calves. Parasitol. Res.

[2] Brunauer M, Roch F.F, Conrady B (2021). Prevalence of worldwide neonatal calf diarrhoea caused by bovine rotavirus in combination with bovine coronavirus, *Escherichia coli* K99 and *Cryptosporidium* spp.:A meta-analysis. Animals.

[3] Thomson S, Hamilton C.A, Hope J.C, Katzer F, Mabbott N.A, Morrison L.J, Innes E.A (2017). Bovine cryptosporidiosis:Impact, host-parasite interaction and control strategies. Vet. Res.

[4] Chalmers R.M, Giles M (2010). Zoonotic cryptosporidiosis in the UK-challenges for control. J. Appl. Microbiol.

[5] Meganck V, Hoflack G, Opsomer G (2014). Advances in prevention and therapy of neonatal dairy calf diarrhoea:A systematic review with emphasis on colostrum management and fluid therapy. Acta Vet. Scand.

[6] Robertson L.J, Campbell A.T, Smith H.V (1992). Survival of *Cryptosporidium parvum* oocysts under various environmental pressures. Appl. Environ. Microbiol.

[7] Harp J.A, Goff J.P (1998). Strategies for the control of *Cryptosporidium parvum* infection in calves. J. Dairy Sci.

[8] Elfstrand L, Lindmark-Månsson H, Paulsson M, Nyberg L, Åkesson B (2002). Immunoglobulins, growth factors and growth hormone in bovine colostrum and the effects of processing. Int. Dairy J.

[9] McGrath B.A, Fox P.F, McSweeney P.L.H, Kelly A.L (2016). Composition and properties of bovine colostrum:A review. Dairy Sci. Technol.

[10] Puppel K, Gołębiewski M, Grodkowski G, Slósarz J, Kunowska-Slósarz M, Solarczyk P, Łukasiewicz M, Balcerak M, Przysucha T (2019). Composition and factors affecting quality of bovine colostrum:A review. Animals.

[11] Pyo J, Pletts S, Romao J (2018). The effects of extended colostrum feeding on gastrointestinal tract growth of the neonatal dairy calf. J. Anim. Sci.

[12] Fischer A.J, Song Y, He Z, Haines D.M, Guan L.L, Steele M.A (2018). Effect of delaying colostrum feeding on passive transfer and intestinal bacterial colonization in neonatal male Holstein calves. J. Dairy Sci.

[13] Pyo J, Fischer A, He Z, Haines D, Guan L, Steele M (2018). PSI-37 the effects of delaying initial colostrum feeding on gastrointestinal tract growth of neonatal bull dairy calves. J. Anim. Sci.

[14] Fujino T, Matsuo T, Okada M, Matsui T (2016). Detection of a small number of *Cryptosporidium parvum* oocysts by sugar flotation and sugar centrifugation methods. J. Vet. Med. Sci.

[15] Henriksen S.A, Pohlenz J.F.L (1981). Staining of cryptosporidia by a modified Ziehl-Neelsen. Acta Vet. Scand.

[16] The Jamovi Project 2021. Jamovi (Version 2.0.0). Computer Software.

[17] Lassen B, Viltrop A, Raaperi K, Järvis T (2009). *Eimeria* and *Cryptosporidium* in Estonian dairy farms in regard to age, species, and diarrhoea. Vet. Parasitol.

[18] Santoro A, Dorbek-Kolin E, Jeremejeva J, Tummeleht L, Orro T, Jokelainen P, Lassen B (2018). Molecular epidemiology of *Cryptosporidium* spp. in calves in Estonia:High prevalence of *Cryptosporidium parvum* shedding and 10 subtypes identified. Parasitology.

[19] Lassen B, Järvis T (2009). *Eimeria* and *Cryptosporidium* in Lithuanian cattle farms. Vet. Zootech.

[20] Robbers L, Jorritsma R, Nielen M, Koets A (2021). A scoping review of on-farm colostrum management practices for optimal transfer of immunity in dairy calves. Front. Vet. Sci.

[21] Derbakova A, Zolovs M, Keidāne D, Šteingolde Ž (2020). Effect of immunoglobulin G concentration in dairy cow colostrum and calf blood serum on *Cryptosporidium* spp. invasion in calves. Vet. World.

[22] Fischer A.J, Hertogs K, Hatew-Chuko B, Steele M.A (2018). Oligosaccharide and IgG concentrations throughout the first week of lactation in multiparous and primiparous Holstein dairy cattle. J. Anim. Sci.

[23] Fischer A.J, Villot C, van Niekerk J.K, Yohe T.T, Renaud D.L, Steele M.A (2019). Invited review:Nutritional regulation of gut function in dairy calves:From colostrum to weaning. Appl. Anim. Sci.

[24] Conneeley M, Berry D.P, Murphy J.P, Lorenz I, Doherty M.L, Kennedey E (2014). Effect of feeding colostrum at different volumes and subsequent number of transition milk feeds on the serum immunoglobulin G concentration and health status of dairy calves. J. Dairy Sci.

[25] Kargar S, Bahadori-Moghaddam M, Ghoreishi S.M, Akhlaghi A, Kanani M, Pazoki A, Ghaffari M.H (2021). Extended transition milk feeding for 3 weeks improves growth performance and reduces the susceptibility to diarrhea in newborn female Holstein calves. Animal.

[26] Blum J.W, Hammon H (2000). Colostrum effects on the gastrointestinal tract, and on nutritional, endocrine and metabolic parameters in neonatal calves. Livest. Prod. Sci.

[27] Meale S.J, Chaucheyras-Durand F, Berends H, Guan L.L, Steele M.A (2017). From pre-to postweaning:Transformation of the young calf's gastrointestinal tract 1. J. Dairy Sci.

[28] Mennerat A, Nilsen F, Ebert D, Skorping A (2010). Intensive farming:evolutionary implications for parasites and pathogens. Evol. Biol.

[29] May R.M, Nowak M.A (1995). Coinfection and the evolution of parasite virulence. Proc. Biol. Sci.

[30] Gandon S, Jansen V.A.A, van Baalen M (2001). Host life history and the evolution of parasite virulence. Evolution.

[31] Li R.W, Connor E.E, Li C, Baldwin Vi R.L, Sparks M.E (2012). Characterization of the rumen microbiota of pre-ruminant calves using metagenomics tools. Environ. Microbiol.

